# Skip metastases in papillary thyroid carcinoma: evidence from a multicenter European retrospective study

**DOI:** 10.3389/fendo.2026.1712563

**Published:** 2026-01-30

**Authors:** Giacomo Di Filippo, Gian Luigi Canu, Giulia Gobbo, Fabio Medas, Leonardo Rossi, Federico Cappellacci, Piermarco Papini, Marinunzia Paternoster, Angeliki Chorti, Ioannis Pliakos, Moysis Moysidis, Giovanni Lazzari, Eleonora Morelli, Dorin Serbusca, Andrea Ruzzenente, Theodosios Papavramidis, Gabriele Materazzi, Pietro Giorgio Calò

**Affiliations:** 1 Endocrine Surgery Unit, Department of Surgery and Oncology, Verona University Hospital, Verona, Italy; 2 Department of Surgical Sciences, University of Cagliari, Cagliari, Italy; 3 Otolaryngology-Head and Neck Surgery Department, University of Verona, Verona, Italy; 4 Endocrine Surgery Unit, University Hospital of Pisa, Pisa, Italy; 5 First Propedeutic Department of Surgery, American Hellenic Educational Progressive Association (AHEPA) University Hospital, Aristotle University of Thessaloniki, Thessaloniki, Greece

**Keywords:** follicular variant, lymph node metastasis, neck dissection, papillary thyroid carcinoma, skip metastasis

## Abstract

**Introduction:**

Papillary thyroid carcinoma (PTC) frequently involves cervical lymph nodes. Lateral nodal involvement without central compartment disease (Skip Metastasis, SM) poses diagnostic and staging challenges. We aimed to characterize clinicopathological features of SM in a large multicenter European cohort comparing SM+ (lateral only) and SM- (central + lateral) disease.

**Materials and methods:**

We conducted a retrospective study across four high-volume European centers (01/2020-12/2022). Adults with histologically proven PTC, confirmed lateral cervical metastases, and both central (level VI) and lateral (levels II–IV) dissections were included. Additional subanalyses among pT1a and cases with >2 central nodes retrieved were employed to control for confounders.

**Results:**

Among 283 patients, 48 (17.0%) were SM +. SM+ patients were older (47 vs 39 years, p=0.006) and had smaller primaries (12 vs 16 mm, p=0.008), fewer microfoci (2 vs 3, p=0.013), fewer lateral nodes retrieved (22 vs 25, p=0.004), fewer positive nodes (2 vs 4, p<0.001), and smaller largest metastatic node (14.5 vs 18.5 mm, p=0.010). Follicular variant was prevalent in SM+ (18.8% vs 4.3%; p=0.001). Features of aggressiveness were less frequent in SM+: multifocality (62.5% vs 74.9%, p=0.016), bilaterality (37.5% vs 52.8%, p=0.024), microscopic ETE (33.3% vs 52.3%, p=0.016), LVI (14.6% vs 47.7%, p<0.001), and extranodal extension (8.3% vs 20.4%, p=0.043). Findings persisted in different subanalyses (>2 central nodes; n=252. pT1a tumors n=73). In multivariable analysis, central nodal yield (OR 1.13, p=0.003) and LVI (OR 6.5, p=0.005) were associated with SM-, whereas follicular variant was inversely associated (OR 0.24, p=0.028).

**Conclusion:**

SM were not uncommon and associated with a less aggressive clinicopathologic profile and increased proportion of follicular variant. While limited central nodal yield may pose false positive risks, key associations persisted after adjustment. Prospective studies are warranted to refine risk stratification and surgical planning.

## 1 Introduction

Papillary thyroid carcinoma (PTC) is the most common form of thyroid cancer and one of the fastest rising malignancies worldwide (1, 2). It accounts for approximately 80-90% of all thyroid cancers and typically follows an indolent course with low mortality (3). According to the 2022 World Health Organization (WHO) classification, PTC can be subclassified in different subtypes based on histomorphologic features irrespective of tumor dimension (4). Despite its generally favorable prognosis, PTC has a strong propensity for regional spread to cervical lymph nodes (5).

Cervical lymph node metastases are detected in a significant proportion of patients at diagnosis. Studies have reported central compartment lymph node involvement in about 20-50% of cases, and lateral cervical lymph node metastases (LLNM) in approximately 20% of cases (6, 7). In PTC, lymphatic spread typically occurs in a stepwise fashion: tumor cells first metastasize to the central neck nodes, then to ipsilateral lateral nodes, followed by contralateral or mediastinal nodes (8). Skip Metastases (SM) refers to a pattern where LLNM occur without involvement of the central compartment. SM are not uncommon in PTC and have been documented across many series with reported incidence ranging from 0.6 to 38% (2, 9–11).

From a clinical standpoint, SM pose a diagnostic and therapeutic challenge. SM may be overlooked if clinicians rely on the absence of central node involvement as a sign that lateral nodes are likely disease-free. Thus, identifying patients at risk for SM preoperatively is crucial to ensure appropriate surgical planning and lymph node dissection extent.

Recent studies have sought to identify clinicopathological factors that predict SM in PTC (12–15). The presence of certain aggressive tumor features has been evaluated with mixed findings - for example, extrathyroidal extension of the tumor has been cited as a possible risk factor in some studies (5, 12, 15), whereas others have not found a significant correlation (16–18). Interestingly, the absence of lymphovascular invasion (LVI) has been associated with a higher likelihood of SM, suggesting that tumors that metastasize directly to the lateral neck might do so without extensive LVI (19). By contrast, factors such as multifocality, tumor bilaterality, and coexistent Hashimoto’s thyroiditis have not shown consistent associations with SM risk (20). The reported inconsistency in risk factors may be due to methodological heterogeneity, including variations in how thoroughly the central compartment is dissected and histopathologically examined.

To date, there are few studies focusing on the pathological characteristics of European patients with SM. We present the findings from a large multicenter study conducted across four European centers that examined a broad cohort of PTC patients who underwent total thyroidectomy with central and lateral neck dissection. In this study, we analyze the clinicopathological characteristics of patients with SM and compare them to those of patients presenting with both CLNM and LLNM.

## 2 Materials and methods

### 2.1 Study design

**Figure 1:**
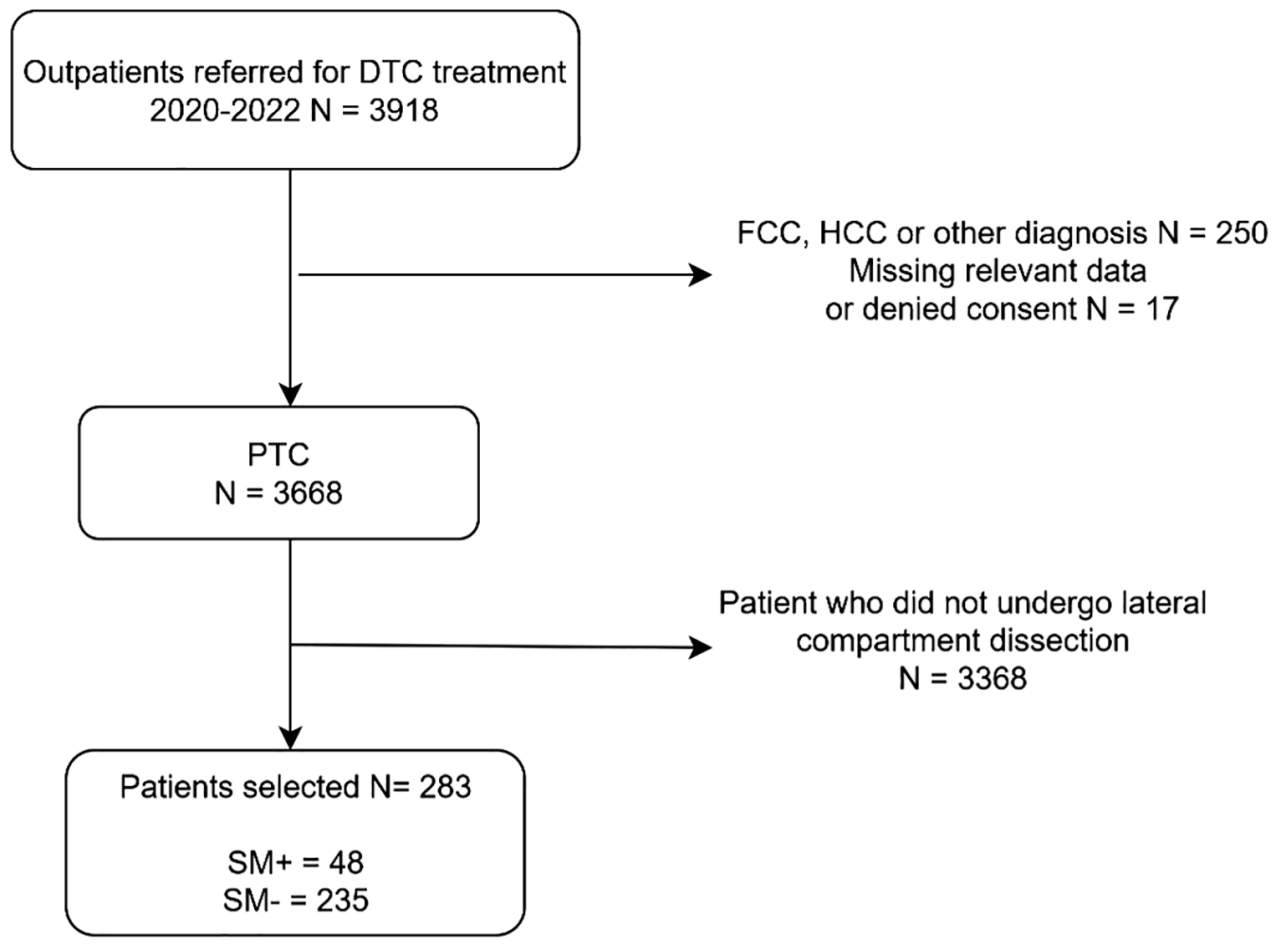
Patients’ selection and inclusion criteria flowchart. DTC, Differentiated thyroid Cancer; FCC, Follicular thyroid cancer; HCC, Hürtle cell thyroid cancer; PTC, Papillary Thyroid Cancer; SM, Skip metastasis.

This retrospective, multicenter, international study was conducted across four European high-volume referral centers for thyroid surgery (Endocrine Surgery Unit, Verona University Hospital; Endocrine Surgery Unit, Cagliari University Hospital; Endocrine Surgery Unit, Pisa University Hospital; Endocrine Surgery Unit, Aristotle University of Thessaloniki), and included patients who underwent surgery for thyroid disease between January 1^st^, 2020, and December 1^st^, 2022 (**Figure 1**). Eligible patients were older than 18 years, had a histopathological diagnosis of PTC with histopathologically confirmed LLNM, and had undergone both central compartment (level VI) and lateral compartment (levels IIa–IV) lymph node dissection. All patients with suspicious lateral cervical lymphadenopathy underwent fine-needle aspiration cytology (FNAC) for cytological confirmation prior to surgery. Patients were excluded if they were younger than 18 years, had a histopathological diagnosis other than PTC, had incomplete or missing data, or had not provided written informed consent for data collection. Clinical data were extracted from the electronic medical records of each institution and included sociodemographic characteristics, clinical findings, surgical details, and histopathological features such as tumor size and location, multifocality, extrathyroidal extension, LVI, and number and distribution of metastatic lymph nodes. Patients were categorized into two groups according to the presence or absence of skip metastases: the SM+ group included patients with LLNM without central compartment involvement, while the SM- group included patients with both lateral and central compartment metastases. A subgroup analysis was performed on patients who had undergone a central compartment dissection that yielded at least three lymph nodes, in order to minimize misclassification due to inadequate sampling, and an additional subgroup analysis was performed on patients with papillary thyroid microcarcinoma (pT1a). Disease was staged according to the AJCC TNM 7th edition classification system. The study was approved by the Ethics Committee of the University of Cagliari and conducted in accordance with the Declaration of Helsinki. Written informed consent for data use was obtained from all patients prior to inclusion.

### 2.2 Statistical analysis

Categorical variables were expressed as absolute frequencies and relative percentages, while continuous variables were described as median values and interquartile ranges. A chi square test was used to compare categorical variables and a Mann Whitney test was employed to compare continuous variables between groups. Subgroup analyses were performed on selected patients based on the relevant aforementioned criteria. A multivariable logistic regression analysis was performed to seek significant predictors of SM, while controlling for confounders. A p value of < 0.05 was considered as statistically significant. Data analysis was performed using SPSS version 25.0 (IBM Corp., Armonk, NY, USA).

## 3 Results

### 3.1 Basic characteristics of the study population

The population’s sociodemographic, clinical and pathological characteristics are summarized in **Table 1**. The study population comprised 283 patients. The median age at the time of surgery was 40 years [30–51]. Females accounted for 59.4% (n=168) of patients. Chronic lymphocytic thyroiditis was present in 39.9% (n=113) of patients, while a history of hyperthyroidism was reported in 4.6% (n=13). Central compartment pathological nodes were observed in 83.0% (n=235, SM-), whereas no metastatic central nodes were identified in 17.0% (n=48, SM+) of patients.

**Table 1:** Sociodemographic, clinical and pathological characteristics of the population.

Variable		N (%); Median (IQR)
Age (years)		40 (30–51)
Gender	Female	168 (59.4%)
	Male	115 (40.6%)
BMI (kg/m^2^)		25 (23–28)
Hyperthyroidism	Yes	13 (4.6%)
	No	270 (95.4%)
Diagnosis	Graves’ Disease	1 (0.4%)
	Intermediate nodule	11 (3.9%)
	Malignancy	267 (94.3%)
	N/MNG	2 (0.7%)
	NMNG	2 (0.7%)
Chronic lymphocytic thyroiditis	Yes	113 (39.9%)
	No	170 (60.1%)
Histological variant	Classic	206 (72.8%)
	Columnar Cell	3 (1.1%)
	Diffuse sclerosing	3 (1.1%)
	Follicular	19 (6.7%)
	Solid	13 (4.6%)
	Tall cell	39 (13.8%)
Aggressive histological variant	Yes	58 (20.5%)
	No	225 (79.5%)
Multifocality	Yes	205 (72.4%)
	No	78 (27.6%)
Maximum neoplasm diameter (mm)		15 (10–23)
Number of microfoci		2 (1–4)
Central compartment lymph nodes (n)		10 (6–15)
Lateral cervical lymph nodes (n)		24 (17–32)
Pathologic lymph nodes in the central compartment (n)		4 (1–9)
Pathologic lateral cervical lymph nodes (n)		3 (2–7)
Pathologic lymph nodes maximum diameter (mm)		17 (12–28)
Bilaterality	Yes	136 (50%)
	No	136 (50%)
Aggressive variant on microfoci	Yes	23 (11.2%)
	No	182 (88.8%)
Surgical margin involvement	Yes	6 (2.1%)
	No	277 (97.9%)
Extrathyroid microscopic infiltration	Yes	139 (49.1%)
	No	144 (50.9%)
Extrathyroid macroscopic infiltration	Yes	29 (10.2%)
	No	254 (89.8%)
Vascular-lymphatic infiltration	Yes	119 (42%)
	No	164 (58%)
Pathological lymph nodes in the central compartment	Yes	235 (83%)
	No	48 (17%)
Extra-nodal infiltration	Yes	53 (18.7%)
	No	230 (81.3%)
ATA risk stratification	High	142 (50.2%)
	Intermediate	141 (49.8%)

IQR, interquartile range; BMI, body mass index; N/MNG, nodular/multinodular goiter; ATA, American Thyroid Association.

**Figure 2:**
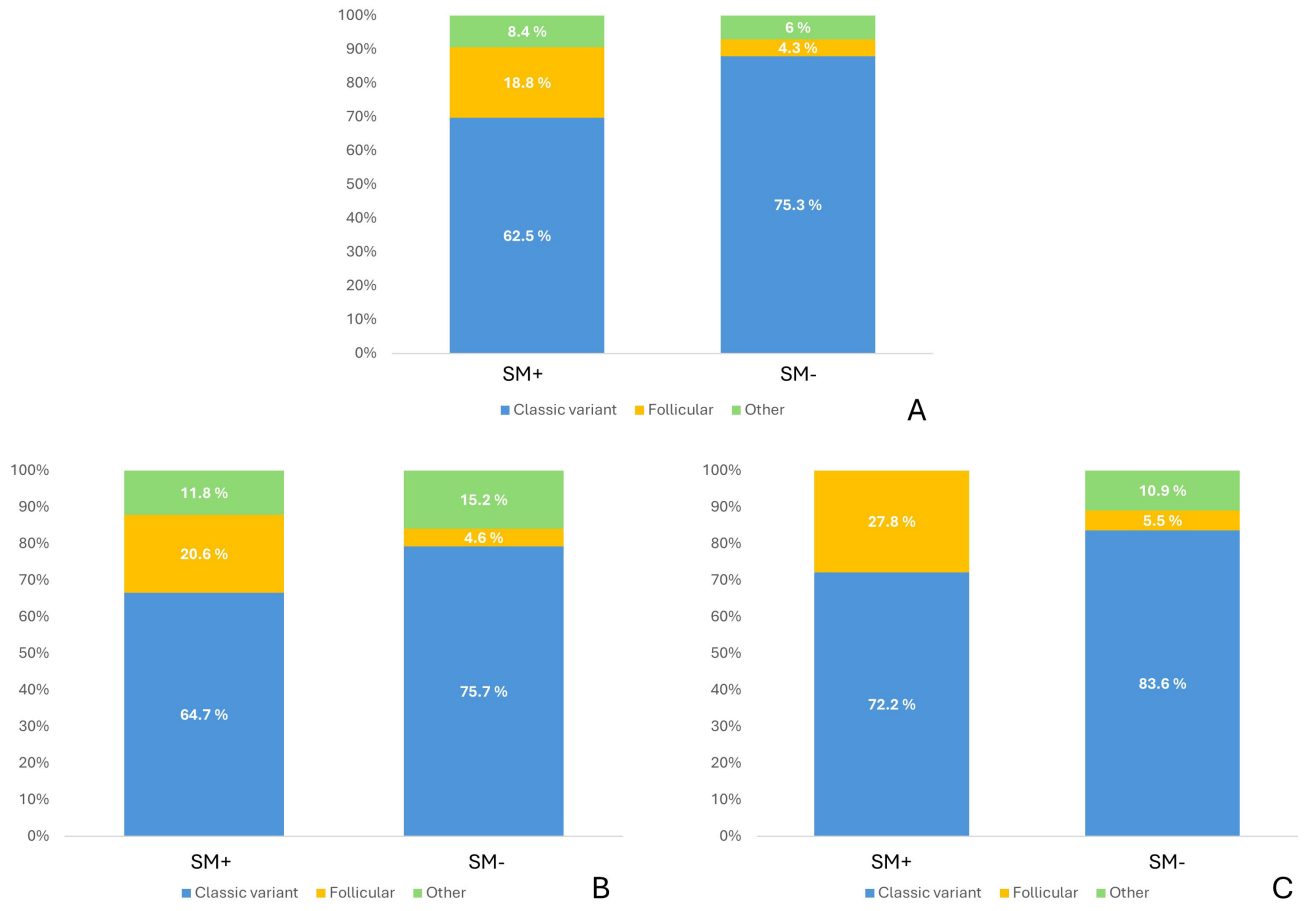
PTC variants distribution among the population. **(A)** whole population, **(B)** patients with central lymph node yield higher than 3, **(C)** patients with pT1a tumors.

Regarding histological subtypes (**Figure 2**), the classic variant was the most frequent (73.1%, n=207), followed by tall cell (13.8%, n=39), follicular (6.7%, n=19), solid (4.2%, n=12), columnar cell (1.1%, n=3), and diffuse sclerosing (1.1%, n=3). The median maximum tumor diameter was 15 mm [10–23]. The median number of central compartment lymph nodes retrieved was 10 [6–15], with a median of 4 [1–9] metastatic central nodes. In the lateral compartment, the median number of lymph nodes removed was 24 [17–32], 3 [2–7] of which were metastatic.

Multifocal disease was documented in 72.4% (n=205), and bilateral disease in 50.0% (n=136) of patients. Microscopic extrathyroidal extension was present in 49.1% (n=139), while macroscopic extension was observed in 10.2% (n=29) of patients. LVI was reported in 42.0% (n=119) of patients. Extranodal extension was identified in 18.7% (n=53) of cases.

### 3.2 Comparison between SM+ and SM- patients

Characteristics of SM+ patients compared to SM- patients are summarized in **Table 2**.

**Table 2:** Sociodemographic, clinical and pathological characteristics. Comparison between SM+ and SM- patients.

Variable		SM +	SM -	p
		N (%), Median (IQR)	N (%), Median (IQR)	
Age (years)		47 (35–61)	39 (29–50)	0.01
Gender	Female	30 (62.5%)	138 (58.7%)	0.63
	Male	18 (37.5%)	97 (41.3%)	
BMI (kg/m^2^)		25 (23–28)	25 (23–28)	0.46
Hyperthyroidism	No	44 (91.7%)	226 (96.2%)	0.17
	Yes	4 (8.3%)	9 (3.8%)	
Preoperative diagnosis	Graves’ Disease	1 (2.1%)	–	0.07
	Intermediate nodule	1 (2.1%)	10 (4.3%)	
	Malignancy	44 (91.7%)	223 (94.9%)	
	N/MNG	2 (4.2%)	2 (0.8%)	
Chronic lymphocytic thyroiditis	No	24 (50%)	146 (62.1%)	0.12
	Yes	24 (50%)	89 (37.9%)	
Histological variant	Classic	30 (62.5%)	176 (74.9%)	<0.0005
	Columnar Cell	2 (4.2%)	1 (0.4%)	
	Diffuse sclerosing	–	3 (1.3%)	
	Follicular	9 (18.8%)	10 (4.3%)	
	Solid	2 (4.2%)	11 (4.7%)	
	Tall cell	5 (10.4%)	34 (14.5%)	
Aggressive histological variant	No	39 (81.3%)	186 (79.1%)	0.74
	Yes	9 (18.8%)	49 (20.9%)	
Multifocality	No	20 (41.7%)	58 (24.7%)	0.02
	Yes	28 (58.3%)	177 (75.3%)	
Bilaterality	No	30 (65.2%)	106 (46.9%)	0.02
	Yes	16 (34.8%)	120 (53.1%)	
Maximum neoplasm diameter (mm)		12 (6–20)	16 (10–25)	0.01
Number of microfoci		2 (1–2)	3 (1–4)	0.01
Central compartment lymph nodes (n)		6 (3–9)	11 (7–16)	<0.0005
Lateral cervical lymph nodes (n)		0	5 (2–10)	<0.0005
Pathologic lymph nodes in the central compartment (n)		22 (13–26)	25 (18–33)	<0.0005
Pathologic lateral cervical lymph nodes (n)		2 (1–2)	4 (2–7)	<0.0005
Pathologic lymph node’s maximum diameter (mm)		14.5 (8–23)	18.5 (13–28)	0.01
Aggressive variant on microfoci	No	27 (96.4%)	155 (87.6%)	0.17
	Yes	1 (3.6%)	22 (12.4%)	
Surgical margin involvement	No	47 (97.9%)	230 (97.9%)	0.99
	Yes	1 (2.1%)	5 (2.1%)	
Extrathyroid microscopic infiltration	No	32 (66.7%)	112 (47.7%)	0.02
	Yes	16 (33.3%)	123 (52.3%)	
Extrathyroid macroscopic infiltration	No	43 (89.6%)	211 (89.8%)	0.97
	Yes	5 (10.4%)	24 (10.2%)	
Vascular-lymphatic infiltration	No	44 (91.7%)	120 (51.1%)	<0.0005
	Yes	4 (8.3%)	115 (48.9%)	
Extra-nodal infiltration	No	44 (91.7%)	186 (79.1%)	0.04
	Yes	4 (8.3%)	49 (20.9%)	
ATA risk stratification	High	12 (25%)	130 (55.3%)	<0.0005
	Intermediate	36 (75%)	105 (44.7%)	

IQR, interquartile range; BMI, body mass index; N/MNG, nodular/multinodular goiter; ATA, American Thyroid Association; SM, Skip Metastases.

Among sociodemographic and clinical variables, SM+ patients were older compared to SM- patients (47 years vs 39 years, p=0.006).

Regarding pathological features, tumors in the SM+ group were smaller than those in the SM- group (median maximum tumor diameter 12 mm vs 16 mm, p=0.008). Patients in the SM+ group had a lower number of tumor foci (median 2 vs 3, p=0.013). Patients with SM had fewer central compartment nodes (6 vs 11, p<0.001) and lateral lymph nodes (22 vs 25, p=0.004) retrieved, and metastatic lateral nodes (2 vs 4, p<0.001). The maximum size of the metastatic lymph node was smaller in SM+ than in SM- patients (14.5 mm vs 18.5 mm, p=0.010).

The histological subtype distribution differed significantly between groups (p=0.001), with SM+ patients being more frequently diagnosed with follicular variant (18.8% vs 4,3%). Patients in the SM+ group had a lower prevalence of multifocal disease (62.5% vs 74.9%, p=0.016) and bilaterality (37.5% vs 52.8%, p=0.024). Microscopic extrathyroidal extension was less frequent in SM+ compared to SM- patients (33.3% vs 52.3%, p=0.016), and LVI was markedly less frequent in SM+ patients (14.6% vs 47.7%, p<0.001). Perinodal extension was also less commonly observed in SM+ compared to SM- patients (8.3% vs 20.4%, p=0.043). Finally, ATA risk stratification categories differed significantly (p<0.001), with SM+ patients being more frequently classified into lower risk categories.

### 3.3 Subgroup analysis: central compartment lymph nodes retrieved > 3

Patients with fewer than 3 central compartment lymph nodes examined were excluded from the original population, resulting in a study population of 252 patients (**Table 3**).

**Table 3:** Sociodemographic, clinical and pathological characteristics. Comparison between SM+ and SM- patients excluding those with central compartment dissection yield <3 lymph nodes.

Variable		SM +	SM -	p
		N (%), Median (IQR)	N (%), Median (IQR)	
Age (years)		51 (39–61)	38 (28–49)	<0.0005
Gender	Female	23 (67.6%)	131 (60.1%)	0.4
	Male	11 (32.4%)	87 (39.9%)	
BMI (kg/m^2^)		26 (23–29)	25 (23–28)	0.24
Hyperthyroidism	No	32 (94.1%)	209 (95.9%)	0.64
	Yes	2 (5.9%)	9 (4.1%)	
Preoperative diagnosis	Graves’ Disease	–	–	0.19
	Intermediate nodule	1 (2.9%)	9 (4.1%)	
	Malignancy	31 (91.2%)	207 (95%)	
	N/MNG	2 (5.8%)	2 (1.0%)	
Chronic lymphocytic thyroiditis	No	14 (41.2%)	133 (61%)	0.03
	Yes	20 (58.8%)	85 (39%)	
Histological variant	Classic	22 (64.7%)	164 (75.2%)	0.001
	Columnar Cell	2 (5.9%)	1 (0.5%)	
	Diffuse sclerosing	–	3 (1.4%)	
	Follicular	7 (20.6%)	10 (4.6%)	
	Solid	1 (2.9%)	11 (5.1%)	
	Tall cell	2 (5.9%)	29 (13.3%)	
Aggressive histological variant	No	29 (85.3%)	174 (79.8%)	0.45
	Yes	5 (14.7%)	44 (20.2%)	
Multifocality	No	13 (38.2%)	52 (23.9%)	0.08
	Yes	21 (61.8%)	166 (76.1%)	
Bilaterality	No	19 (55.9%)	97 (46.2%)	0.3
	Yes	15 (44.1%)	113 (53.8%)	
Maximum neoplasm diameter (mm)		10 (6–16)	15 (10–24)	<0.0005
Number of microfoci		1 (1–2)	3 (1–4)	0.01
Central compartment lymph nodes (n)		7 (5–9)	12 (8–16)	<0.0005
Lateral cervical lymph nodes (n)		23 (15–25)	25 (18–34)	0.01
Pathologic lymph nodes in the central compartment (n)		–	6 (3–10)	<0.0005
Pathologic lateral cervical lymph nodes (n)		2 (1–2)	4 (2–7)	<0.0005
Pathologic lymph node’s maximum diameter (mm)		13.5 (8–21)	19 (13–28)	<0.0005
Aggressive variant on microfoci	No	21 (100%)	145 (87.3%)	0.08
	Yes	–	21 (12.7%)	
Surgical margin involvement	No	33 (97.1%)	214 (98.2%)	0.67
	Yes	1 (2.9%)	4 (1.8%)	
Extrathyroid microscopic infiltration	No	24 (70.6%)	106 (48.6%)	0.02
	Yes	10 (29.4%)	112 (51.4%)	
Extrathyroid macroscopic infiltration	No	31 (91.2%)	197 (90.4%)	0.9
	Yes	3 (8.8%)	21 (9.6%)	
Vascular-lymphatic infiltration	No	31 (91.2%)	111 (50.9%)	<0.0005
	Yes	3 (8.8%)	107 (49.1%)	
Extra-nodal infiltration	No	32 (94.1%)	172 (78.9%)	0.04
	Yes	2 (5.9%)	46 (21.1%)	
ATA risk stratification	High	7 (20.6%)	120 (55.0%)	<0.0005
	Intermediate	27 (79.4%)	98 (45%)	

IQR, interquartile range; BMI, body mass index; N/MNG, nodular/multinodular goiter; ATA, American Thyroid Association; SM, Skip Metastases.

Among clinical variables, age remained significantly higher in SM+ compared with SM- patients (51 years vs 38 years, p<0.001).

Regarding pathological features, tumors in the SM+ group were smaller than in the SM- group (10 mm vs 15 mm, p<0.001). The number of tumor foci was also lower in SM+ patients (1 vs 3, p=0.011). Patients with SM had fewer central lymph nodes and lateral lymph nodes retrieved (7 vs 12, p<0.001 and 23 vs 25, p=0.010, respectively), and fewer metastatic lateral nodes (2 vs 4, p<0.001). The maximum diameter of the metastatic lymph node was smaller in SM+ compared with SM- patients (13.5 mm vs 19.0 mm, p=0.004).

Chronic lymphocytic thyroiditis was more frequent in SM+ compared to SM- patients (58.8% vs 39%, p=0.029). Histological subtype distribution differed significantly between groups (p=0.001), with SM+ still showing a higher proportion of follicular variant compared to SM- patients (20.6% vs 4.6%). Microscopic extrathyroidal extension was less frequent in SM+ than in SM- patients (27.8% vs 49.4%, p=0.017). Lymphovascular invasion was markedly lower in SM+ patients (8.3% vs 46.3%, p<0.001). Extranodal extension was also less common in SM+ compared with SM- patients (5.6% vs 20.4%, p=0.036). Finally, ATA risk stratification categories differed significantly, with SM+ patients more frequently classified into lower risk categories (Intermediate 79.4% vs 45% p<0.001).

### 3.4 Subgroup analysis: pT1a patients

In a further subgroup analysis, we included only patients with papillary thyroid microcarcinoma (pT1a). The resulting subgroup comprised 73 patients (SM+: n=18; SM-: n=55) (**Table 4**).

**Table 4:** Sociodemographic, clinical and pathological characteristics. Comparison between SM+ and SM- patients excluding those with maximum tumor diameter > 10 mm.

Variable		SM +	SM -	
		N (%), Median (IQR)	N (%), Median (IQR)	p
Age (years)		55 (39–62)	42 (34–49)	0.02
Gender	Female	12 (66.7%)	35 (63.6%)	0.82
	Male	6 (33.3%)	20 (36.4%)	
BMI (kg/m^2^)		25 (23–27)	25 (21–29)	0.8
Hyperthyroidism	No	16 (88.9%)	53 (96.4%)	0.23
	Yes	2 (11.1%)	2 (3.6%)	
Preoperative diagnosis	Grave’s Disease	–	–	0.22
	Intermediate nodule	–	2 (3.6%)	
	Malignancy	16 (88.9%)	52 (94.5%)	
	N/MNG	2 (11.2%)	1 (1.8%)	
Chronic lymphocytic thyroiditis	No	6 (33.3%)	36 (65.5%)	0.02
	Yes	12 (66.7%)	19 (34.5%)	
Histological variant	Classic	13 (72.2%)	46 (83.6%)	0.04
	Columnar Cell	–	–	
	Diffuse sclerosing	–	–	
	Follicular	5 (27.8%)	3 (5.5%)	
	Solid	–	4 (7.3%)	
	Tall cell	–	2 (3.6%)	
Aggressive histological variant	No	18 (100%)	49 (89.1%)	0.15
	Yes	–	6 (10.9%)	
Multifocality	No	7 (38.9%)	10 (18.2%)	0.07
	Yes	11 (61.1%)	45 (81.8%)	
Bilaterality	No	9 (50%)	26 (48.1%)	0.9
	Yes	9 (50%)	28 (51.9%)	
Maximum neoplasm diameter (mm)		6 (2–10)	7 (5–9)	0.58
Number of microfoci		2 (1–3)	2 (1–4)	0.23
Central compartment lymph nodes (n)		7 (5–9)	10 (7–17)	0.01
Lateral cervical lymph nodes (n)		23 (16–25)	23 (16–32)	0.34
Pathologic lymph nodes in the central compartment (n)		–	4 (2–7)	<0.0005
Pathologic lateral cervical lymph nodes (n)		1 (1–2)	3 (2–5)	<0.0005
Pathologic lymph node’s maximum diameter (mm)		13.5 (8–16)	15 (10–23)	0.09
Aggressive variant on microfoci	No	11 (100%)	41 (91.1%)	0.31
	Yes	–	4 (8.9%)	
Surgical margin involvement	No	18 (100%)	54 (98.2%)	0.57
	Yes	–	1 (1.8%)	
Extrathyroid microscopic infiltration	No	15 (83.3%)	39 (70.9%)	0.3
	Yes	3 (16.7%)	16 (29.1%)	
Extrathyroid macroscopic infiltration	No	17 (94.4%)	53 (96.4%)	0.72
	Yes	1 (5.6%)	2 (3.6%)	
Vascular-lymphatic infiltration	No	17 (94.4%)	42 (76.4%)	0.09
	Yes	1 (5.6%)	13 (23.6%)	
Extra-nodal infiltration	No	17 (94.4%)	50 (90.9%)	0.64
	Yes	1 (5.6%)	5 (9.1%)	
ATA risk stratification	High	2 (11.1%)	16 (29.1%)	0.13
	Intermediate	16 (88.9%)	39 (70.9%)	

IQR, interquartile range; BMI, body mass index; N/MNG, nodular/multinodular goiter; ATA, American Thyroid Association; SM, Skip Metastases.

Among sociodemographic variables, age was higher in SM+ than in SM- patients (55 years vs 42 years, p=0.016). Chronic lymphocytic thyroiditis was more frequent in SM+ than SM- patients (66.7% vs 34.5%, p=0.017).

The number of central compartment lymph nodes retrieved was lower in SM+ than SM- patients (7 vs 10, p=0.008), with fewer metastatic lateral nodes (1 vs 3, p<0.001). The distribution of histological variants also differed between groups (p=0.039) with a higher prevalence of follicular variant among SM+ patients compared to SM- patients (27.8% vs 5.5%).

### 3.5 Multivariable analysis

A multivariable logistic regression model was built to identify significant predictors of SM while accounting for clinically relevant confounders in the total study population (n=283).

The result of this model is summarized in **Table 5**.

**Table 5:** Multivariable logistic regression analysis with predictors of SM- (stepwise disease).

Variable	B	OR	95% CI	p
Maximum neoplasm diameter (mm)	0,035	1,035	(0,98 – 1,09)	0.184
Central compartment lymph nodes (n)	0,127	1,135	(1,04 – 1,24)	0.003
Classic variant (reference)				0.089
Follicular variant	-1,415	0,243	(0,07 – 0,86)	0.028
Other variants	-0,318	0,728	(0,22 – 2,36)	0.597
Multifocality	0,762	2,142	(0,63 – 7,31)	0.224
Bilaterality	-0,457	0,633	(0,19 – 2,08)	0.452
Chronic lymphocytic thyroiditis	-0,623	0,536	(0,23 – 1,25)	0.149
Vascular-lymphatic infiltration	1,875	6,521	(1,76 – 24,16)	0.005
Extrathyroid microscopic infiltration	0,442	1,557	(0,62 – 3,92)	0.348

OR, odds ratio; CI, confidence interval; SM, Skip Metastases.

Independent factors associated with SM- were the number of central lymph nodes retrieved (OR 1.13, p=0.003), follicular variant of PTC (OR 0.24, p=0.028) and LVI (OR 6.5, p=0.005).

## 4 Discussion

To the best of our knowledge, our study is among the few multicentric European analyses focusing on SM in PTC. Most prior reports have been single-center studies with relatively limited patient cohorts. The rate of SM observed in our series falls within the broad range reported in the literature (12, 13, 18, 21), reinforcing the knowledge that SM, while not the predominant pattern of spread, are not uncommon in PTC. In our study population, patients with SM had less aggressive pathological features compared to those with stepwise nodal spread. This is reflected by smaller tumors, lower frequencies of tumor multifocality, bilateral disease, extrathyroidal extension, LVI, and extranodal extension found in the SM+ group. These patterns support the hypothesis that SM may arise from less aggressive tumors via a distinct anatomic lymphatic pathway of spread, as substantiated by prior reports (11, 17). For instance, Machens et al. noted that SM are often seen in less aggressive forms of PTC, implying that patients with isolated lateral nodal spread often lack other high-risk features (22). More recently, Yoon et al. found that in papillary microcarcinoma patients with lateral node involvement, those with SM had significantly lower rates of bilaterality and multifocality than those with stepwise spread, and smaller lateral tumor burden (23). Such tumors might rely on specific anatomical lymphatic routes rather than aggressive local invasion, in line with findings by Lei et al. (24).

A relevant finding of our study was the higher prevalence of the follicular variant of PTC (FVPTC) found in SM+ patients. This finding remained significant even when adjusting for tumor size and for the extent of central node dissection. Tumor biology influences nodal spread even in other types of neoplasms (25–27) and subtype-specific behavior in PTC is well documented (28–32). Li et al. compared outcomes of FVPTC vs classic PTC in 799 patients and found that FVPTC had significantly less extrathyroidal extension, fewer lymph node metastases, and lower rates of capsular invasion than classic PTC (33). On the other hand, there have been also reports of unusual metastatic patterns in FVPTC, indicating that this variant may have unique tumor features and behavior (34, 35). Taken together, a potential overlap of biological behavior and pathological characteristics may be hypothesized for FVPTC and SM. However, current evidence is not yet sufficient to conclusively prove variant-specific lymphatic spread pathways. Further dedicated studies are needed to validate this potential association between FVPTC histology and SM. A significant relationship between lower central lymph node yield and SM+ was highlighted in our study population. Indeed, multiple studies suggest that a limited dissection of the central compartment can misclassify nodal spread, erroneously labeling stepwise nodal spread as SM+ (8, 22). Zhao et al., for instance, demonstrated that an increased number of central lymph nodes dissected was inversely associated with the occurrence of SM (2). For cN0 PTC patients, some authors have suggested that removing ≥3 central lymph nodes can be considered an adequate sampling for staging purposes, with even higher yields desirable for larger tumors to increase the sensitivity of the dissection (36). We agree with the consensus that central compartment dissection should be as complete as oncologically appropriate, especially in patients at higher risk. Although a low central compartment nodal yield may partly account for apparent SM, key tumor characteristics remain associated with SM even after adjustment for nodal yield.

### 4.1 Strengths and limitations

This study has 2 main limitations. First, its retrospective design inherently carries risks of selection bias and unmeasured confounding. Second, ultrasonographic data were not uniformly available, precluding analysis of tumor location within the thyroid, a factor that has been repeatedly associated with skip metastases in prior literature. Consequently, we could not assess or adjust for this potentially important predictor in our study population.

However, the study has some notable strengths. It is a multicenter European investigation from four high-volume endocrine surgery units with a substantial sample size, enhancing external validity within European practice. Additionally, the collection of multiple clinicopathological features allowed for potential confounders adjustment in regression analyses. Together, these features help providing an estimate of the clinicopathological profile associated with skip metastases and aid in contextualizing this pattern of spread.

## 5 Conclusion

In our study population, SM were associated with several pathological markers of lower aggressiveness when compared to stepwise nodal disease. A multifactorial explanation may be hypothesized including a distinct anatomic lymphatic route, different variant-related biologic behavior given the higher proportion of follicular variant, and, at least in part, limited central compartment dissection which could overestimate SM due to partial misclassification. However, such results should be interpreted with caution given the discussed limitations. Future studies, ideally prospective and with standardized surgical and pathological protocols, are warranted to validate our observations and to better elucidate the underlying mechanisms of SM in PTC.

## Data Availability

The data that support the findings of this study are available from the corresponding author, upon reasonable request.
