# Supplementary figures and images for: Skip metastases in papillary thyroid carcinoma: evidence from a multicenter European retrospective study
Source: Front Endocrinol (Lausanne). 2026 Jan 30;17:1712563. doi: 10.3389/fendo.2026.1712563 (PMC12900679; doi:10.3389/fendo.2026.1712563)

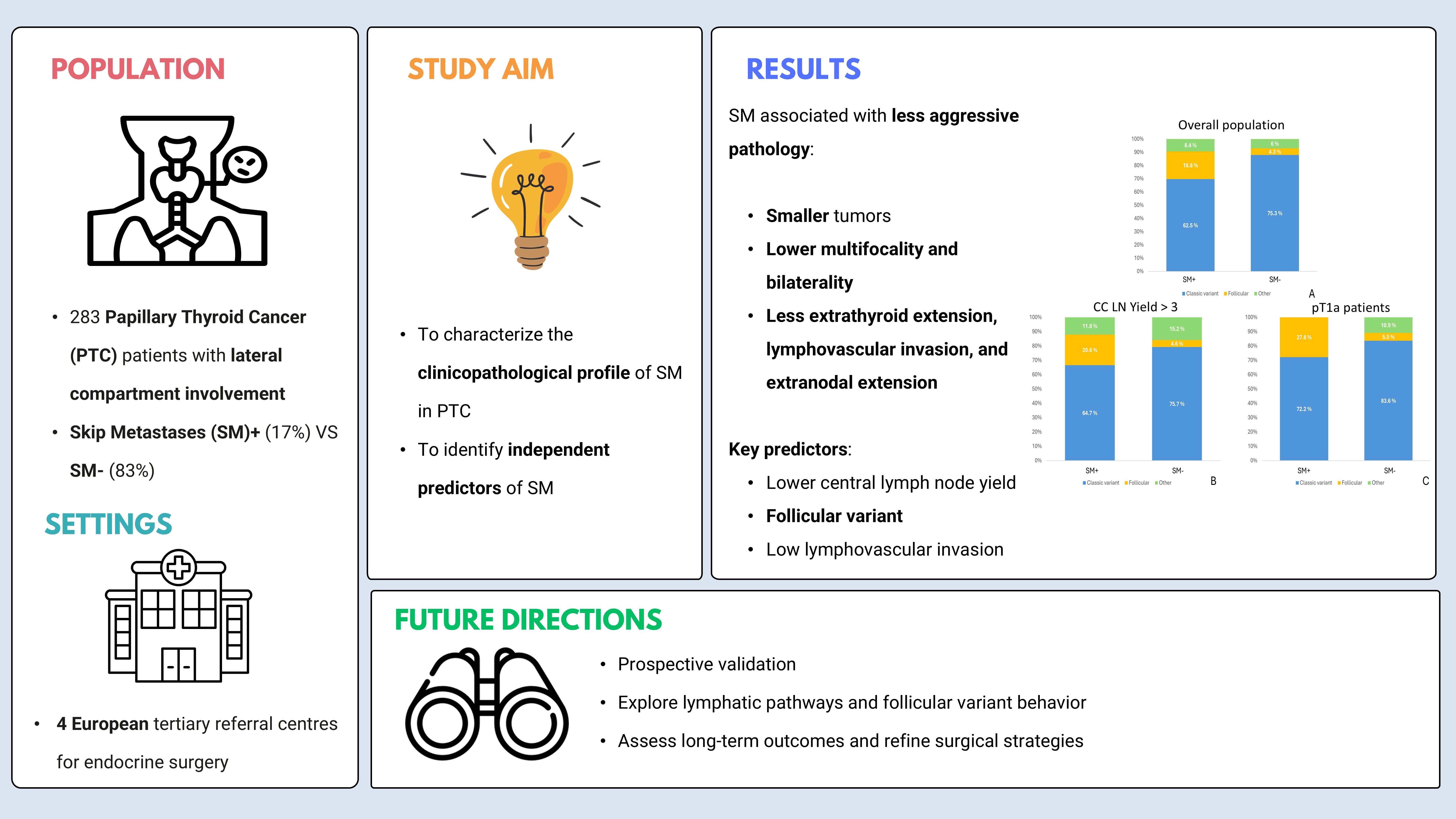

Supplement: Supplementary file 1 [file Image1.jpeg]
